# Supplementary material for: Translatome of dorsal striatum parvalbumin interneurons revisited: insights across diverse experimental paradigms
Source: Front Cell Neurosci. 2025 Oct 15;19:1648461. doi: 10.3389/fncel.2025.1648461 (PMC12568463; doi:10.3389/fncel.2025.1648461)
Supplement: Supplementary file 1 [file Data_Sheet_1.pdf]

Translatome of Dorsal Striatum Parvalbumin interneurons revisited: insights across diverse experimental paradigms

Claire Naon[1*], Laia Castell[2*], Steeve Thirard[3], Maria Moreno[3], Stéphanie Rialle[4], Eva Goetz[1], Eloi Casals[5,6], Angelina Rogliardo[1], Marta Gut[5,6], Anna Esteve-Codina[5,6], Albert Quintana[7,8], Federica Bertaso[1], Emmanuel Valjent[1*], Laura Cutando[7,8*].

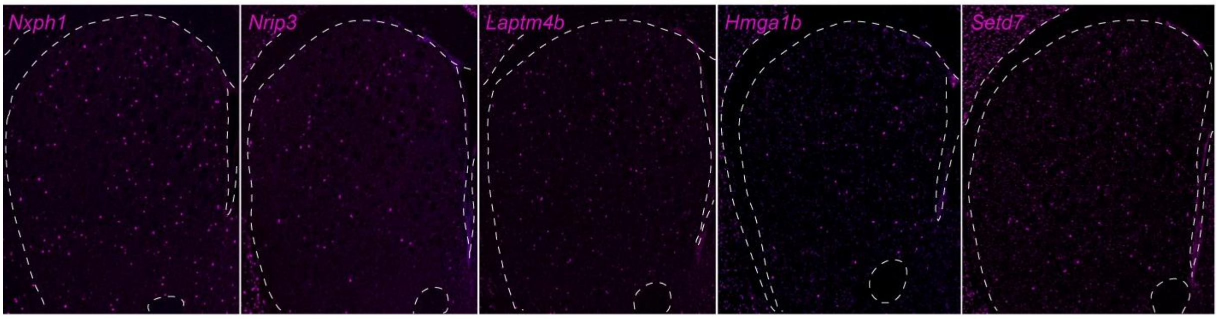

Supplementary Figure 1: **Expression of genes identified by RNAseq into the DS of C57BL/6 mice.** *In situ* hybridization example images for 5 representative genes expressed in PV interneurons (from the Allen Brain Atlas).

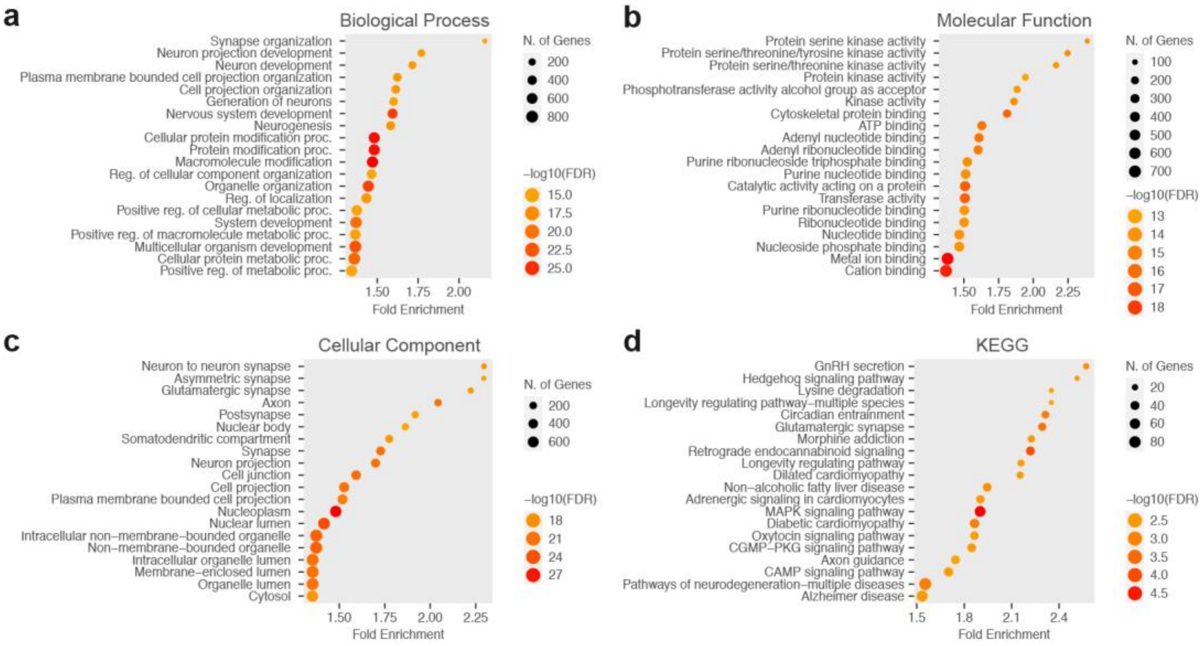

Supplementary Figure 2: **Gene ontology analysis of DS PV interneurons enriched genes.** Gene ontology analysis of the (**a**) biological processes, (**b**) molecular functions, (**c**) cellular component and (**d**) Kyoto Encyclopedia of Genes and Genomes (KEGG) pathways.

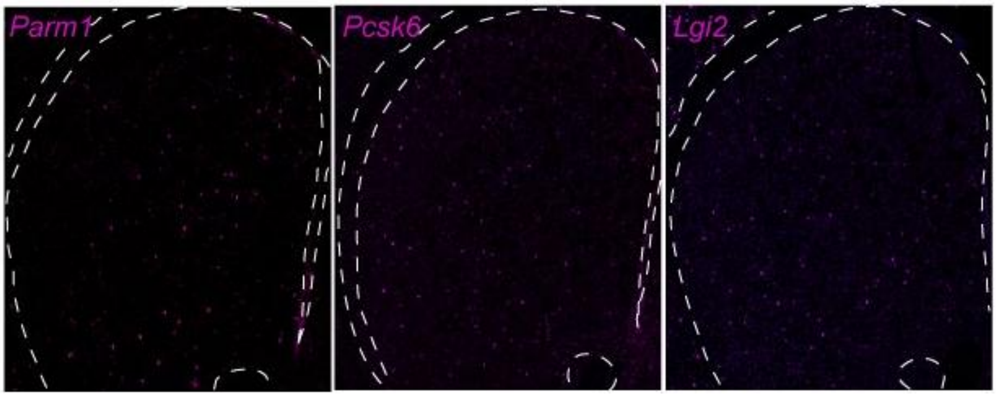

Supplementary Figure 3: **Expression of genes identified by RNAseq into the DS of C57BL/6 mice.** *In situ* hybridization example images for 3 representative extracellular matrix (ECM) genes expressed in PV interneurons (from the Allen Brain Atlas).

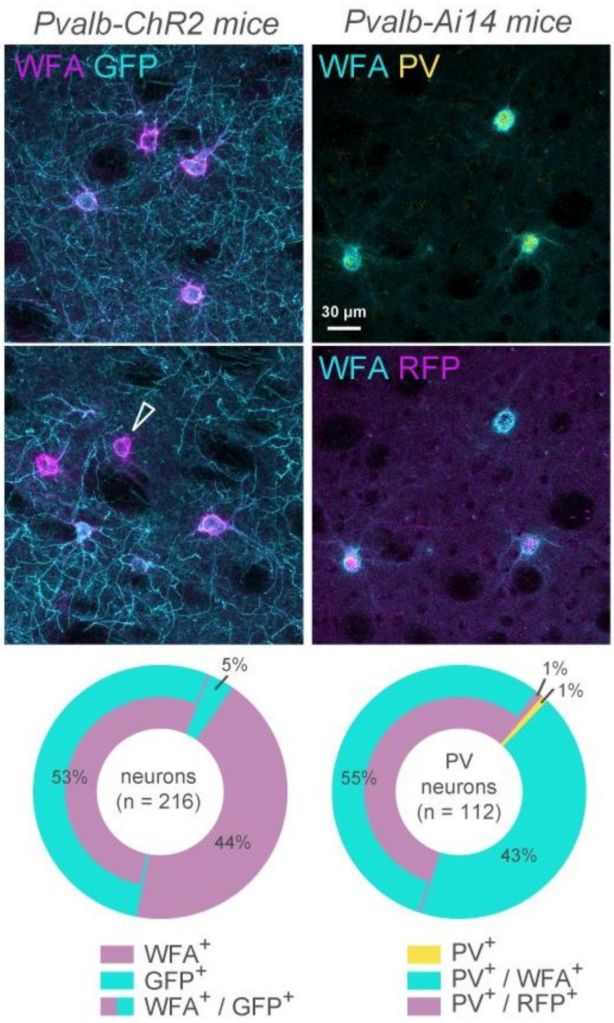

Supplementary Figure 4: Perineuronal nets identified using Wisteria floribunda agglutinin (WFA) in the two different *Pvalb* reporter mouse lines used in this study: *Pvalb-ChR2* (**left**) and *Pvalb-Ai14* (**right**). The presence of ECM is visible around the majority of PV-positive cells and only a minority of PV-negative cells (arrow). Doughnut charts depict the percentage of co-labelling for the different reporters.

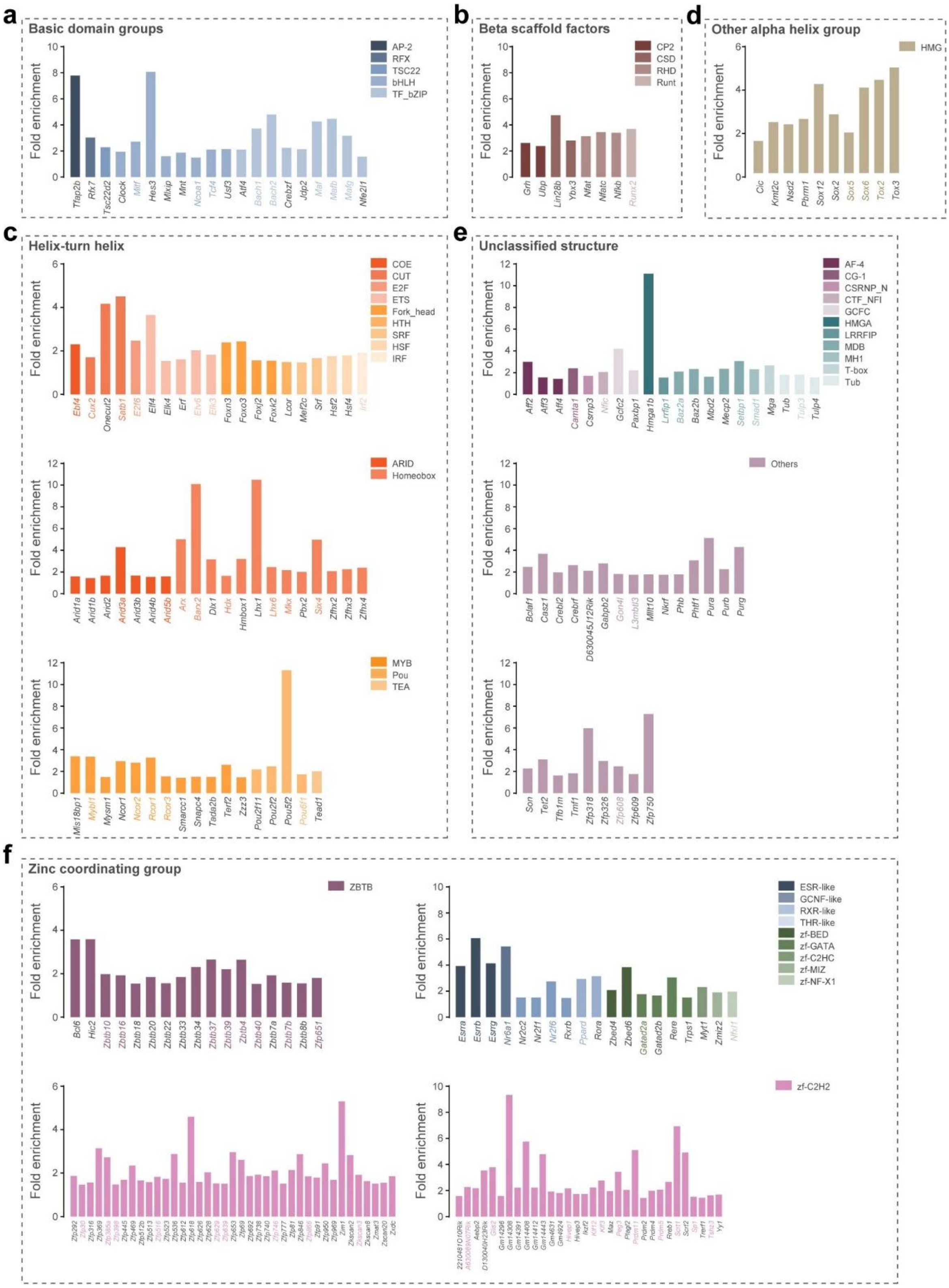

Supplementary Figure 5: **Distribution of DS PV interneurons enriched genes among 6 core families of transcription.** PV-enriched genes found in (**a**) the basic domain groups, (**b**) the beta

scaffold factors, (**c**) the helix-turn helix group, (**d**) the other alpha helix groups, (**e**) the unclassified structure and (**f**) the zinc coordinating group. Color gradient identified enriched transcripts within subfamilies among the 6 core families. Gene names in colors are also found enriched in the Acb.

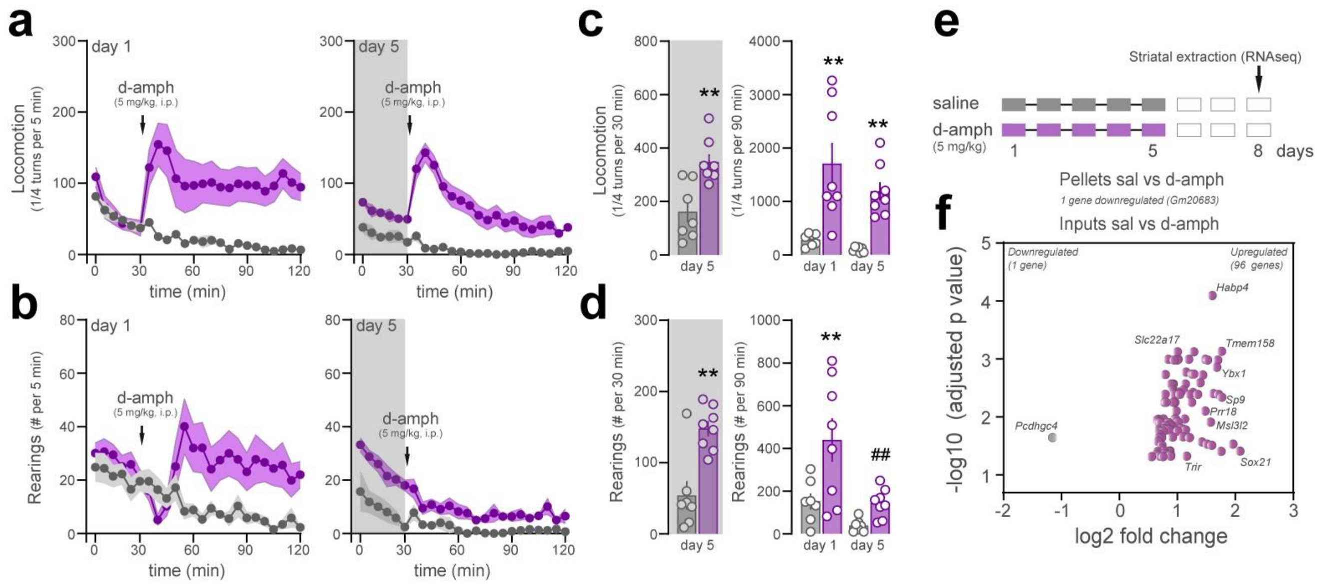

Supplemental Figure 6: **Limited gene expression changes induced in PV interneurons by repeated psychostimulant administration.** (**a**) Locomotor activity (top) and (**b**) rearings (bottom) induced by d-amphetamine on day 1 (left) and day 5 (right) of administration (5 mg/kg i.p., purple; saline controls, grey). (**c-d**) Summary histograms showing locomotion (**c**) and rearings (**d**) on day 5 before drug administration (left, grey area) and comparison between day 1 and day 5 locomotion and rearings (right). (**e**) Administration protocol showing RNAseq analysis being performed 8 days after the first d-amphetamine (or saline) injection. (**f**) Volcano plot of significantly enriched or de-enriched genes in total striatal tissue (input) upon d-amphetamine treatment. One downregulated and 96 upregulated transcripts were identified. Of notice, only one gene (*Gm20683*) was found downregulated by d-amphetamine specifically in PV interneurons (pellet). ** $p < 0.01$ saline vs. d-amph; ## < 0.01 day 1 vs. day 5 (detailed statistical analysis in Supplemental Table 4: 6a-d).

Supplementary Table 4:

Figure	Groups (n: number of mice)	Statistical Analysis
6a – Lever press	Master standard (n=17) Yoked standard (n=17) Master high palatable (n=17) Yoked high palatable (n=17)	**FR1: Three-way ANOVA repeated measures**<br>Time $F_{(4, 256)} = 46.24$, $p < 0.0001$<br>Diet $F_{(1, 64)} = 8.117$, $p = 0.0059$<br>Group $F_{(1, 64)} = 46.40$, $p < 0.0001$<br>Interaction time x diet $F_{(4, 256)} = 6.670$, $p < 0.0001$<br>Interaction time x group $F_{(4, 256)} = 39.07$, $p < 0.0001$<br>Interaction diet x group $F_{(1, 64)} = 5.465$, $p = 0.0225$<br>Interaction factor time x diet x group $F_{(4, 256)} = 5.952$, $p = 0.0001$<br><br>**FR5: Three-way ANOVA repeated measures**<br>Time $F_{(3, 192)} = 27.25$, $p < 0.0001$<br>Diet $F_{(1, 64)} = 23.33$, $p < 0.0001$<br>Group $F_{(1, 64)} = 170.4$, $p < 0.0001$<br>Interaction time x diet $F_{(3, 192)} = 15.2$, $p < 0.0001$<br>Interaction time x group $F_{(3, 192)} = 18.25$, $p < 0.0001$<br>Interaction diet x group $F_{(1, 64)} = 20.99$, $p < 0.0001$<br>Interaction factor time x diet x group $F_{(3, 192)} = 20.99$, $p < 0.0001$<br><br>**FR5 - AL: Three-way ANOVA repeated measures**<br>Time $F_{(5, 320)} = 15.11$, $p < 0.0001$<br>Diet $F_{(1, 64)} = 15.06$, $p = 0.0002$<br>Group $F_{(1, 64)} = 175.5$, $p < 0.0001$<br>Interaction time x diet $F_{(5, 320)} = 2.452$, $p = 0.0336$<br>Interaction time x group $F_{(5, 320)} = 16.34$, $p < 0.0001$<br>Interaction diet x group $F_{(1, 64)} = 7.808$, $p = 0.0069$<br>Interaction factor time x diet x group $F_{(5, 320)} = 2.772$, $p = 0.0181$
6a - Pellets	Master standard (n=17) Yoked standard (n=17) Master high palatable (n=17) Yoked high palatable (n=17)	**FR1: Three-way ANOVA repeated measures**<br>Time $F_{(4, 256)} = 141.0$, $p < 0.0001$<br>Diet $F_{(1, 64)} = 12.33$, $p = 0.0008$<br>Group $F_{(1, 64)} = 0.01067$, $p = 0.9181$<br>Interaction time x diet $F_{(4, 256)} = 8.861$, $p < 0.0001$<br>Interaction time x group $F_{(4, 256)} = 0.04188$, $p = 0.9967$<br>Interaction diet x group $F_{(1, 64)} = 0.01125$, $p = 0.9159$<br>Interaction factor time x diet x group $F_{(4, 256)} = 0.04800$, $p = 0.9956$<br><br>**FR5: Three-way ANOVA repeated measures**<br>Time $F_{(3, 192)} = 25.73$, $p < 0.0001$<br>Diet $F_{(1, 64)} = 40.79$, $p < 0.0001$<br>Group $F_{(1, 64)} = 0.01523$, $p = 0.9022$<br>Interaction time x diet $F_{(3, 192)} = 25.46$, $p < 0.0001$<br>Interaction time x group $F_{(3, 192)} = 0.09958$, $p = 0.9602$<br>Interaction diet x group $F_{(1, 64)} = 0.0001119$, $p = 0.9916$

		Interaction factor time x diet x group $F_{(3, 192)} = 0.05905$, p = 0.9811<br><br>**FR5 - AL: Three-way ANOVA repeated measures**<br>Time $F_{(5, 315)} = 55.80$, p < 0.0001<br>Diet $F_{(1, 64)} = 39.15$, p < 0.0001<br>Group $F_{(1, 64)} = 1.439$, p = 0.2348<br>Interaction time x diet $F_{(5, 315)} = 1.202$, p = 0.3079<br>Interaction time x group $F_{(5, 315)} = 0.5560$, p = 0.7337<br>Interaction diet x group $F_{(1, 64)} = 0.01724$, p = 0.8959<br>Interaction factor time x diet x group $F_{(5, 315)} = 0.4012$, p = 0.8479
6c - Visits	Master standard (n=17) Yoked standard (n=17) Master high palatable (n=17) Yoked high palatable (n=17)	**FR1: Three-way ANOVA repeated measures**<br>Time $F_{(4, 200)} = 16.50$, p < 0.0001<br>Diet $F_{(1, 50)} = 15.95$, p = 0.0002<br>Group $F_{(1, 50)} = 0.6361$, p = 0.4289<br>Interaction time x diet $F_{(4, 200)} = 6.198$, p = 0.0001<br>Interaction time x group $F_{(4, 200)} = 3.362$, p = 0.0109<br>Interaction diet x group $F_{(1, 50)} = 1.586$, p = 0.2138<br>Interaction factor time x diet x group $F_{(4, 200)} = 0.8447$, p = 0.4984<br><br>**FR5: Three-way ANOVA repeated measures**<br>Time $F_{(3, 144)} = 7.016$, p = 0.0002<br>Diet $F_{(1, 50)} = 18.38$, p < 0.0001<br>Group $F_{(1, 50)} = 1.210$, p = 0.2765<br>Interaction time x diet $F_{(3, 144)} = 2.670$, p = 0.0498<br>Interaction time x group $F_{(3, 144)} = 7.722$, p < 0.0001<br>Interaction diet x group $F_{(1, 50)} = 1.852$, p = 0.1797<br>Interaction factor time x diet x group $F_{(3, 144)} = 2.839$, p = 0.0401<br><br>**FR5 - AL: Three-way ANOVA repeated measures**<br>Time $F_{(5, 240)} = 12.38$, p < 0.0001<br>Diet $F_{(1, 48)} = 13.18$, p = 0.0007<br>Group $F_{(1, 48)} = 0.09346$, p = 0.7611<br>Interaction time x diet $F_{(5, 240)} = 0.3991$, p = 0.8492<br>Interaction time x group $F_{(5, 240)} = 0.8006$, p = 0.5502<br>Interaction diet x group $F_{(1, 48)} = 0.2303$, p = 0.6335<br>Interaction factor time x diet x group $F_{(5, 240)} = 0.9701$, p = 0.4368
S6a	Saline (n = 7) d-amphetamine (n = 8)	**Day 1: Two-way ANOVA**<br>Time $F_{(23, 312)} = 1.346$, p = 0.1365<br>Treatment $F_{(1, 312)} = 174$, p < 0.0001<br>Interaction $F_{(23, 312)} = 2.495$, p = 0.0003<br><br>**Day 5: Two-way ANOVA**<br>Time $F_{(23, 312)} = 7.082$, p < 0.0001

		Treatment $F_{(1, 312)}$ = 408.0, p < 0.0001<br>Interaction $F_{(23, 312)}$ = 5.223, p < 0.0001
S6b	Saline (n = 7)<br>d-amphetamine (n = 8)	**Day 1: Two-way ANOVA**<br>Time $F_{(23, 312)}$ = 1.524, p = 0.0606<br>Treatment $F_{(1, 312)}$ = 68.96, p < 0.0001<br>Interaction $F_{(23, 312)}$ = 1.934, p = 0.007<br><br>**Day 5: Two-way ANOVA**<br>Time $F_{(23, 312)}$ = 13.00, p < 0.0001<br>Treatment $F_{(1, 312)}$ = 154.1, p < 0.0001<br>Interaction $F_{(23, 312)}$ = 1.711, p = 0.0238
S6c	Saline (n = 7)<br>d-amphetamine (n = 8)	**Conditioned reponses: Student t-test**<br>t_{13} = 3.871, p = 0.019<br><br>**Sensitization day1 vs day 5: Two-way ANOVA**<br>Day $F_{(1, 26)}$ = 2.516, p = 0.1248<br>Treatment $F_{(1, 26)}$ = 30.36, p < 0.0001<br>Interaction $F_{(1, 26)}$ = 0.5015, p = 0.4852
S6d	Saline (n = 7)<br>d-amphetamine (n = 8)	**Conditioned responses: Student t-test**<br>t_{13} = 4.249, p = 0.009<br><br>**Sensitization day1 vs day 5: Two-way ANOVA**<br>Day $F_{(1, 26)}$ = 12.28, p = 0.0017<br>Treatment $F_{(1, 26)}$ = 11.55, p = 0.0022<br>Interaction $F_{(1, 26)}$ = 2.229, p = 0.1774
